# Integrating hyperspectral features and physiological parameters for quantitative estimation of fig rust severity

**DOI:** 10.3389/fpls.2026.1930419

**Published:** 2026-09-11

**Authors:** Yu Li, Xiangyu Chen, Yiren Din, Haiyan Liu, Yu Wang, Bobo Cao, Xingchen Fang, Yingnan Wang, Ze Zhang

**Affiliations:** 1 Xinjiang Production and Construction Crops Oasis Eco-Agriculture Key Laboratory, College of Agriculture, Shihezi University, Shihezi, China; 2 Kizilsu Kyrgyz Autonomous Prefecture Forestry Administration Station, Artush, China

**Keywords:** disease severity estimation, fig, hyperspectral reflectance spectroscopy, physiological parameters multi-source information fusion, rust disease

## Abstract

**Introduction:**

Fig rust caused by Phakopsora nishidana is a destructive foliar disease that impairs leaf photosynthetic capacity and seriously endangers fig yield and production stability. Traditional visual diagnosis of fig rust severity relies on manual observation, which is labor-intensive, highly subjective, and incapable of large-scale, quantitative disease evaluation. Although hyperspectral remote sensing has been widely adopted for plant disease monitoring, few studies have integrated hyperspectral reflectance features and leaf physiological parameters for quantitative estimation of fig rust severity, restricting the accurate and refined monitoring of this disease.

**Methods:**

This study took leaf lesion coverage as a continuous quantitative indicator of fig rust severity, and collected hyperspectral reflectance data (400–1000 nm) and leaf physiological parameters from 336 fig leaf samples covering five disease severity levels. We systematically analyzed the variation characteristics of leaf physiological indexes under different disease degrees, and comprehensively compared the performance of different spectral preprocessing methods, feature wavelength selection algorithms, and machine learning regression models for fig rust severity prediction.

**Results:**

With the aggravation of fig rust infection, leaf chlorophyll and water content decreased significantly, while anthocyanin content increased remarkably; flavonol content fluctuated across different disease levels and only presented a weak correlation with lesion coverage. The combination of Savitzky–Golay smoothing and first derivative (SG+FD) was identified as the optimal spectral preprocessing method, and the competitive adaptive reweighted sampling (CARS) algorithm outperformed the successive projections algorithm (SPA) in feature wavelength screening. The pure spectral CARS-SVR model yielded an R² of 0.849 and an RMSE of 4.393, while the SVR model based solely on physiological parameters achieved an R² of 0.778 and an RMSE of 5.372. The fusion SVR model integrating spectral and physiological information obtained the best prediction performance, with R², RMSE, and RPD reaching 0.924, 3.067, and 3.641, respectively. Ablation analysis confirmed that chlorophyll content was the dominant factor improving model prediction accuracy. Additionally, model prediction reliability declined under high disease severity, with the severity of heavily infected leaves generally underestimated, showing uneven model performance across different infection levels.

**Discussion:**

The fusion of hyperspectral and leaf physiological data can effectively improve the accuracy and stability of quantitative fig rust severity estimation. The proposed method provides a reliable leaf-level technical solution for precise monitoring of fig rust, and offers valuable theoretical and methodological references for the intelligent quantitative evaluation of crop foliar diseases.

## 1 Introduction

The fig (*Ficus carica L.*) is an economically important fruit tree valued for its high nutritional content and strong ecological adaptability. Large-scale cultivation has been established in Xinjiang, Shandong, and southern China (Li D. et al., 202). However, as cultivation has expanded, diseases have become a major constraint on fig production. Among them, rust, caused by the plant pathogenic fungus *Phakopsora nishidana*, is one of the most destructive diseases affecting figs. The pathogen primarily infects leaves, initially producing yellowish-brown spore clusters pustules on the undersides. As the disease progresses, the lesions spread across the leaf surface, leading to premature defoliation and a substantial reduction in photosynthetic efficiency. In severe cases, fruit quality declines and yield losses can exceed 30%, posing a significant challenge to the sustainable development of the fig industry (Boshoff et al., 2022; Padamsee and McKenzie, 2024). Traditional rust assessment relies on manual visual scoring, which is labor-intensive, time-consuming, and subjective, making it difficult to achieve objective and quantitative evaluation of disease severity. Although laboratory-based measurements can provide accurate information on disease-induced physiological changes, they are often time-consuming and unsuitable for rapid disease assessment. Therefore, an objective and quantitative method for estimating fig rust severity at the leaf level is needed.

Hyperspectral capture continuous spectral information across the visible–near-infrared (VIS–NIR) and short-wave infrared (SWIR) regions and is highly sensitive to disease-induced changes in leaf cell structure, chlorophyll content, water status, and other physiological characteristics (Bin et al., 2013; Jiangsheng et al., 2019; Li et al., 2021). In recent years, hyperspectral reflectance has been widely applied to crop disease detection and severity assessment. For example, Velásquez et al. (2024) identified sensitive wavelengths associated with disease-related physicochemical characteristics and developed a robust classification model capable of accurately detecting *Glomerella anthracis* infection within 48 h after inoculation. Xuan et al. (2022) reported that a PLS-DA model based on fused hyperspectral data achieved a validation accuracy of 91.4%. Kurihara and Yamana (2022) developed a normalized difference spectral index-based model that distinguished healthy and diseased tissues with overall accuracy exceeding 0.94 using the 660–690 nm and 720–760 nm spectral bands. Chen et al. (2020) proposed a spectral indicator based on the 730 and 790 nm wavelengths to distinguish healthy peanut plants from those infected with bacterial wilt. Similarly, Deng et al. (2019) combined entropy distance-ordered reverse selection with machine learning algorithms to enable non-destructive detection of citrus huanglongbing and accurate classification of healthy, symptomatic, and asymptomatic plants. These studies demonstrate the potential of hyperspectral reflectance for plant disease detection. However, its application to fig rust remains largely unexplored.

Moreover, most existing studies have focused on disease classification and disease severity assessment using discrete labels such as “healthy,” “mild,” “moderate,” and “severe” as prediction targets. Although this classification approach can reflect different stages of disease development, it cannot accurately characterize the continuous progression of disease, thereby limiting the precise assessment of disease severity. In addition, most disease monitoring models rely primarily on spectral information or image features. These models mainly analyze the external characteristics of disease and overlook the influence of endogenous biochemical components on the spectral response to disease (Bock et al., 2010). Consequently relying on spectral information alone makes it difficult to comprehensively characterize the mechanisms underlying disease development, thereby limiting the accuracy and robustness of model predictions. In recent years, hyperspectral technology has been widely used to estimate key physiological indicators, such as chlorophyll content (Singhal et al., 2019; Sudu et al., 2022), anthocyanin content (Luo et al., 2022; Li et al., 2023), and leaf water content (Liu et al., 2018; Zou et al., 2022), and has gradually been applied to disease monitoring. Liu et al. (2023) proposed a hyperspectral imaging-based method to non-destructively monitor ApMV-infected apple leaves by predicting leaf chlorophyll content as a quantitative indicator of disease severity. Cheng et al. (2026) integrated vegetation indices, texture features, and plant traits into a partial least squares regression (PLSR) model for monitoring powdery mildew in rubber trees, achieving an R^2^ of 0.794 and an RMSE of 7.991. Park et al. (2023) combined hyperspectral imaging with partial least squares discriminant analysis to establish a non-destructive method for the early detection of ginseng root rot, demonstrating that short-wave infrared spectroscopy can accurately identify the disease and reveal its effects on plant physiology and biochemistry. Although this approach has been applied to the monitoring of various crop diseases, systematic research integrating hyperspectral features with physiological and biochemical parameters for fig rust remain limited.

To address this gap, we investigated fig leaves with different levels of rust infection. First, a continuous disease assessment system was established based on the proportion of leaf lesion area. Second, hyperspectral data were integrated with key physiological indicators, including chlorophyll, anthocyanin, and leaf water contents, to analyze the physiological changes and spectral response characteristics of leaves under disease stress. Third, the Successive Projections Algorithm (SPA) and Competitive Adaptive Reweighted Sampling (CARS) were used to select sensitive wavelengths, which were combined with PLSR, SVR, and XGBoost to develop quantitative models for disease severity estimation. Finally, a multi-source information fusion model integrating hyperspectral and physiological parameters was constructed to evaluate its potential of fig rust severity.

## 2 Materials and methods

**Figure 1:**
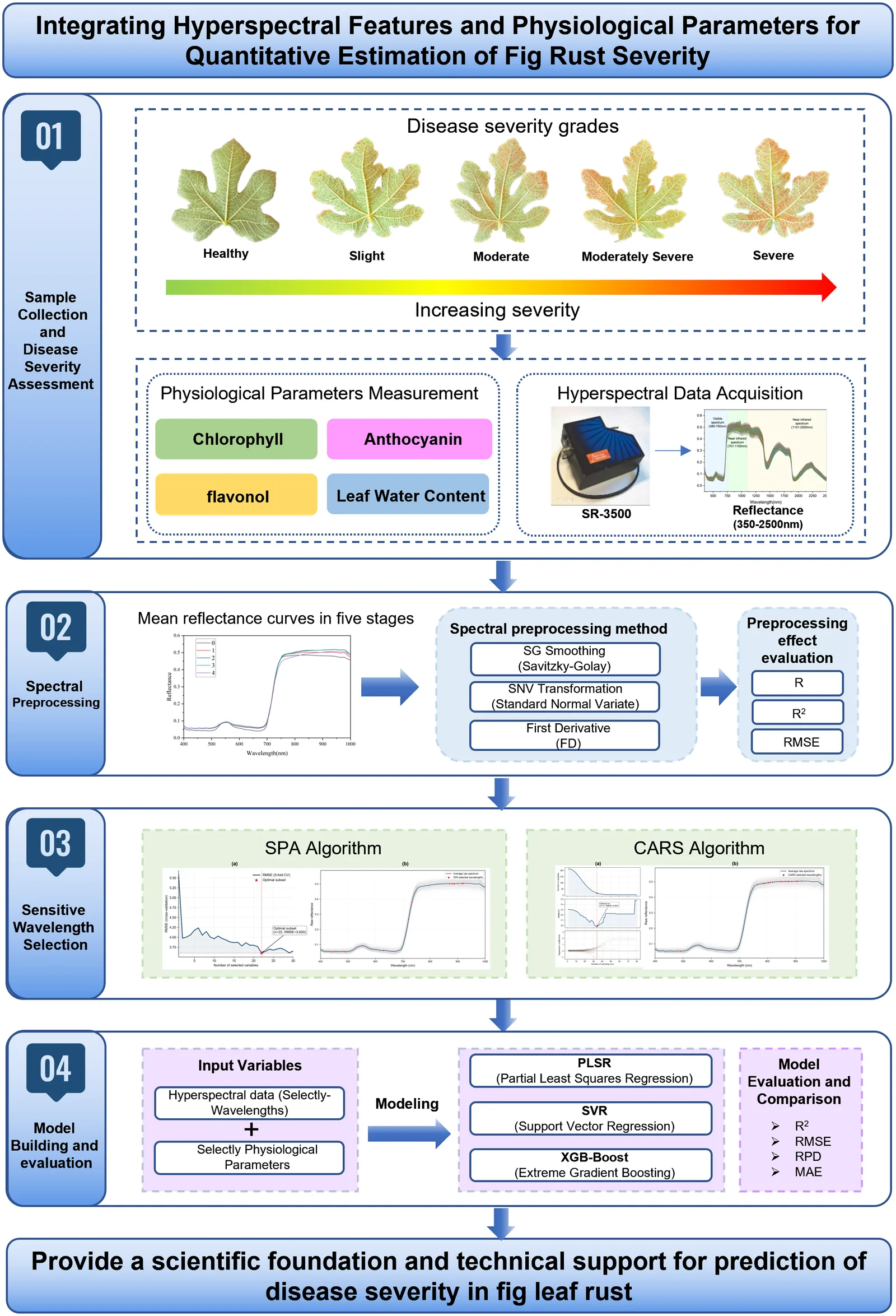
Technical workflow of the study.

**Figure 1** illustrates the technical workflow of this study, which consists of four stages: data acquisition and disease severity classification, spectral preprocessing, feature band selection, and model development and evaluation.

### 2.1 Test materials

**Figure 2:**
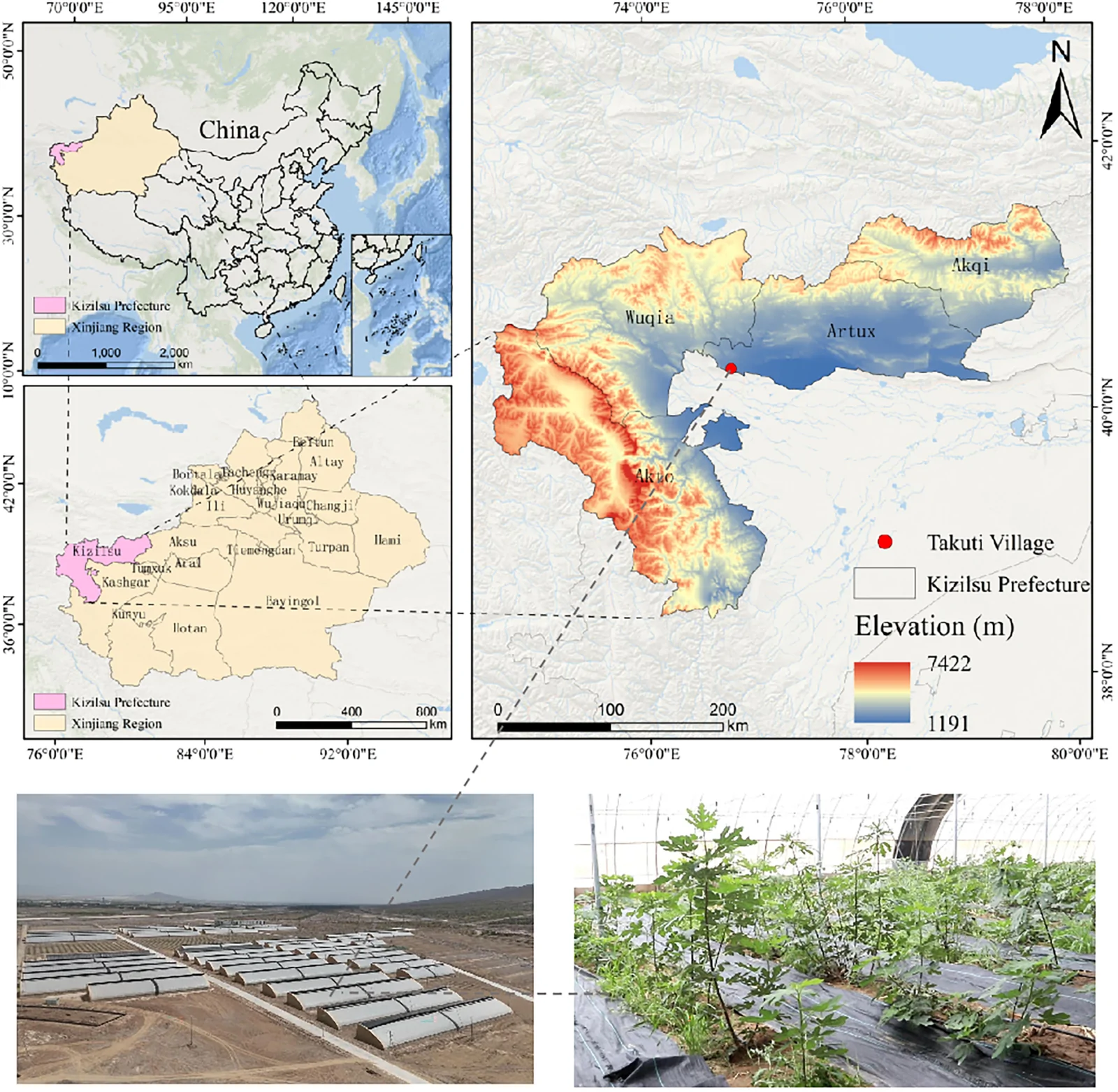
Overview of the study site.

The fig leaves used in this study were collected from April 9 to April 13, 2026, from a fig greenhouse located in the Takuiti Village Tertiary Industry Integration Demonstration Park, Kizilsu Kyrgyz Autonomous Prefecture, Xinjiang, China (39.35°N, 76.02°E). An overview of the experimental site is shown in **Figure 2**. A total of 390 leaves representing different rust severity levels were collected from approximately 100 trees during the disease epidemic period, including healthy, mild, moderate, moderately severe, and severe samples. Approximately three to four functional leaves were collected from each tree from different canopy positions. All samples were collected between 9:00 and 11:00 a.m. on sunny days to minimize environmental variation. After collection, leaf samples were labeled and transported to the laboratory for image acquisition, physiological measurements, and hyperspectral analysis. Leaves with obvious mechanical damage, severe curling, or abnormal spectral responses were excluded during quality control. Abnormal spectra were defined as those exhibiting saturated reflectance values or discontinuous spectral curves after white-reference correction. Finally, 336 valid samples were retained for subsequent analysis and model development.

### 2.2 Classification of disease severity

**Figure 3:**
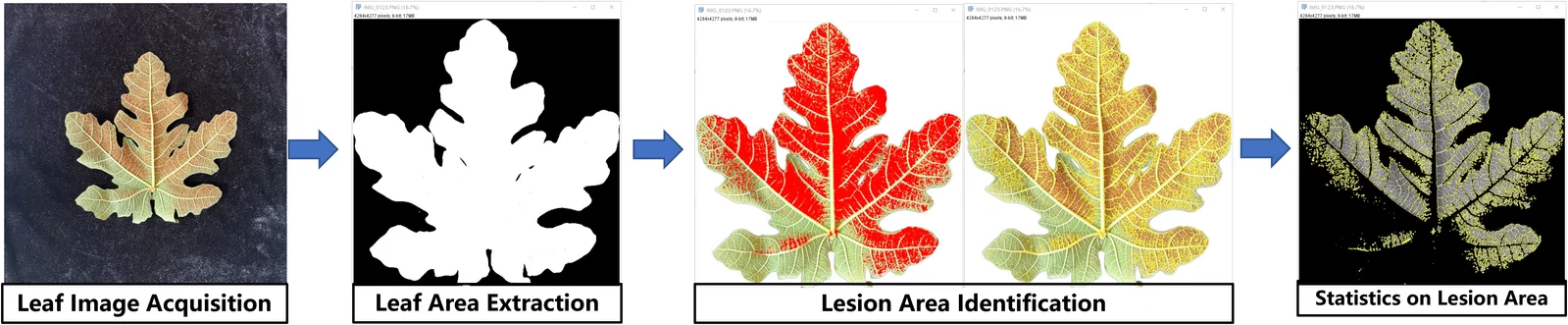
Workflow for lesion coverage extraction using ImageJ.

Disease severity was quantified based on the percentage of leaf area covered by rust lesions. Because rust pustules are predominantly distributed on the abaxial surface of fig leaves, RGB images of the abaxial leaf surfaces were captured using a digital camera positioned vertically above the leaf samples. The camera height, shooting angle, and imaging conditions were kept constant during image acquisition. The acquired images were imported into ImageJ software (National Institutes of Health, USA). Lesion segmentation was performed using the Color Threshold function in the Hue–Saturation–Brightness (HSB) color space. Threshold values were adjusted individually for each image according to lesion color characteristics and image brightness. Segmentation quality was assessed by visually comparing the extracted lesion masks with the original images to ensure accurate identification of rust lesions while minimizing omission and over-segmentation errors. The complete image processing procedure is illustrated in **Figure 3**. The lesion area and total leaf area were then calculated separately, and lesion coverage (DP, %) was calculated as Equation 1:

(1) $$DP=\frac{A_{d}}{A_{l}}\times 100\%$$

where *DP* is the lesion coverage (%), *A_d_* is the lesion area, and *A_l_* is the total leaf area.

The disease severity levels were determined according to lesion coverage thresholds combined with field observations. The thresholds of 5%, 15%, and 25% represented progressive stages from initial lesion appearance to lesion expansion and severe infection. Detailed classification criteria are shown in **Table 1**.

**Table 1:** Criteria for grading fig rust severity.

Disease severity level	RGB image	Lesion coverage	Disease description
0	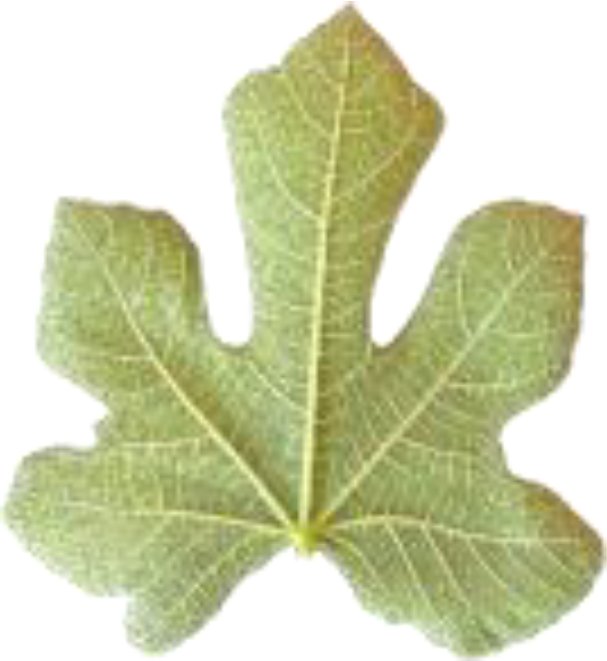	DP=0%	The leaves show no visible disease symptoms and are free of infection.
1	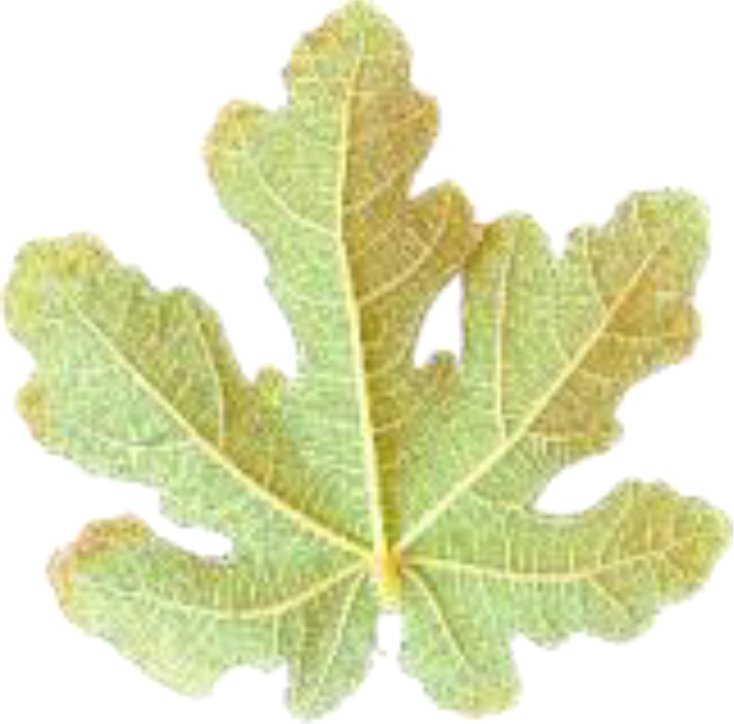	0%<DP ≤ 5%	A few small lesions are present on the upper leaf surface, with a small number of spore clusters on the underside, indicating mild infection.
2	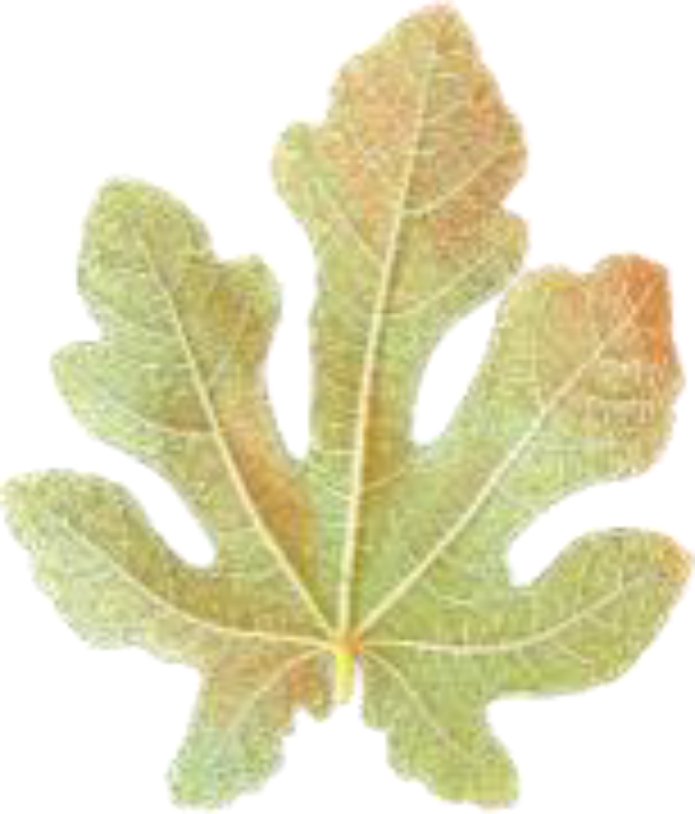	5%<DP ≤ 15%	The number of lesions on the upper leaf surface increases, with localized clusters of spores on the underside indicating moderate infection.
3	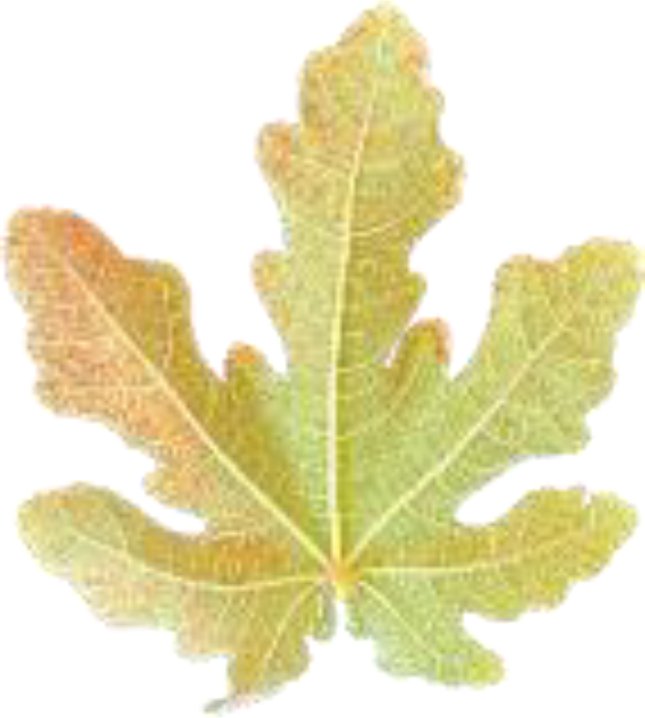	15%<DP ≤ 25%	Lesions spread extensively across the upper leaf surface and large clusters of spores are present on the underside, indicating advanced disease development.
4	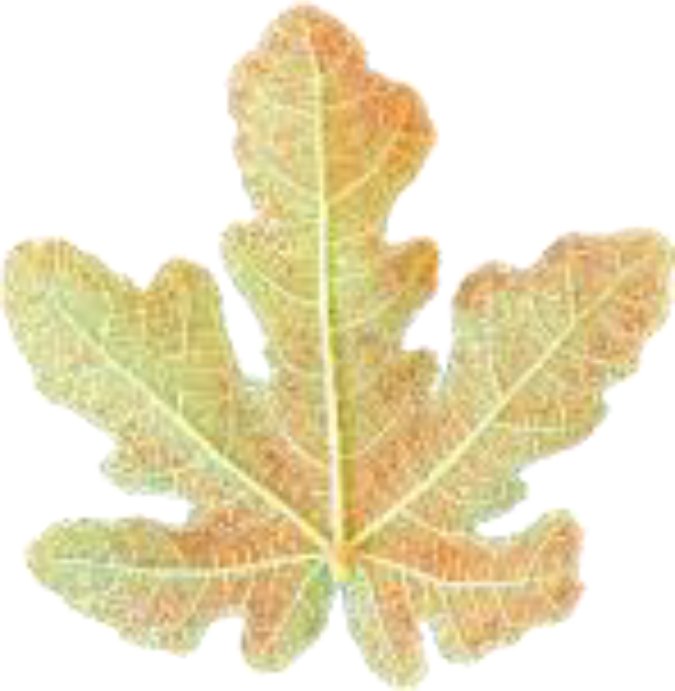	DP>25%	The upper leaf surface exhibits extensive browning, while the underside is covered with dense spore clusters, indicating severe infection.

### 2.3 Acquisition of physiological indicators and hyperspectral data

#### 2.3.1 Measurement of physiological parameters

**Figure 4:**
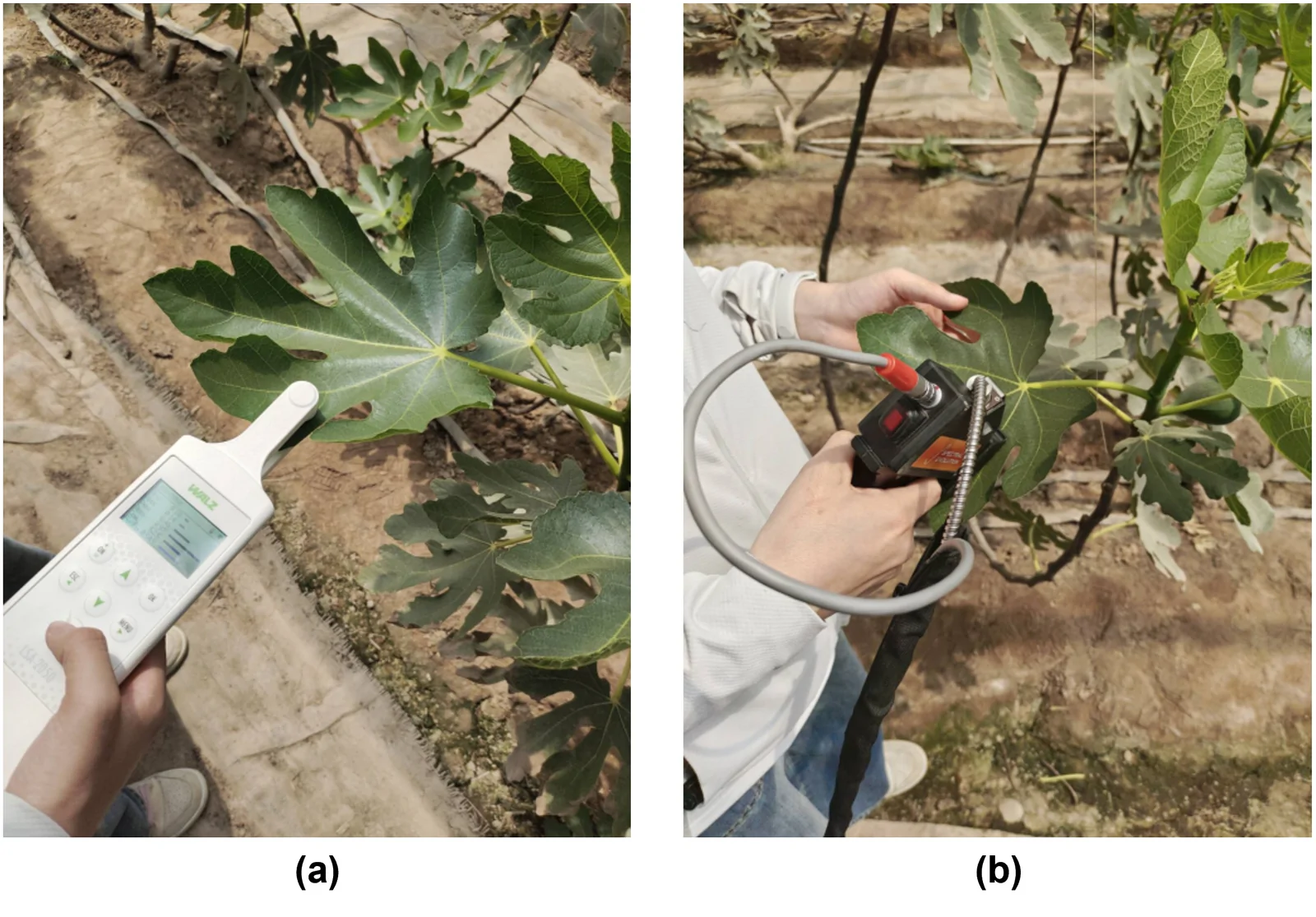
Schematic diagram of physiological parameter and hyperspectral data acquisition for fig leaves. **(a)** Data acquisition using the LSA-2050; **(b)** data acquisition using the SR3500.

The LSA-2050 Plant Polyphenol Chlorophyll Fluorometer (Heinz Walz GmbH, Effeltrich, Germany) was used for non-destructive characterization of leaf pigment status status (**Figure 4a**). According to the manufacturer’s manual (Heinz Walz GmbH, 2024), the instrument employs fluorescence excitation at different wavelength ranges to characterize the UV/visible radiation-screening properties of leaf tissues. In this study, chlorophyll, anthocyanin, and flavonol-related parameters were selected for physiological assessment. Chlorophyll was expressed as an area-based estimate (nmol cm^-2^ and µg cm^-2^), whereas flavonol and anthocyanin were represented as dimensionless fluorescence-derived indices. For each leaf, measurements were taken while avoiding the main veins. Five measurement points were randomly selected, and each point was measured 2 times. The mean value of the repeated measurements was used as the representative pigment-related measurement for each leaf. Leaf water content was determined using the oven-drying method. First, the fresh weight (FW) of each leaf was measured. The samples were then heated at 105 °C for 30 min, after which the temperature was reduced to 85 °C and maintained until a constant weight was reached. The dry weight (DW) was then recorded. Leaf water content was calculated as follows (Equation 2):

(2) $$LWC=\frac{FW-DW}{FW}\times 100\%$$

where LWC is the leaf water content (%), *FW* is the fresh weight, (g), and *DW* is the dry weight (g).

These physiological parameters were selected to characterize disease-induced changes in leaf pigment status and water status. The resulting physiological measurements were subsequently integrated with hyperspectral information to investigate spectral response mechanisms and support multi-source information fusion for quantitative assessment of fig rust severity.

#### 2.3.2 Hyperspectral data acquisition

**Figure 5:**
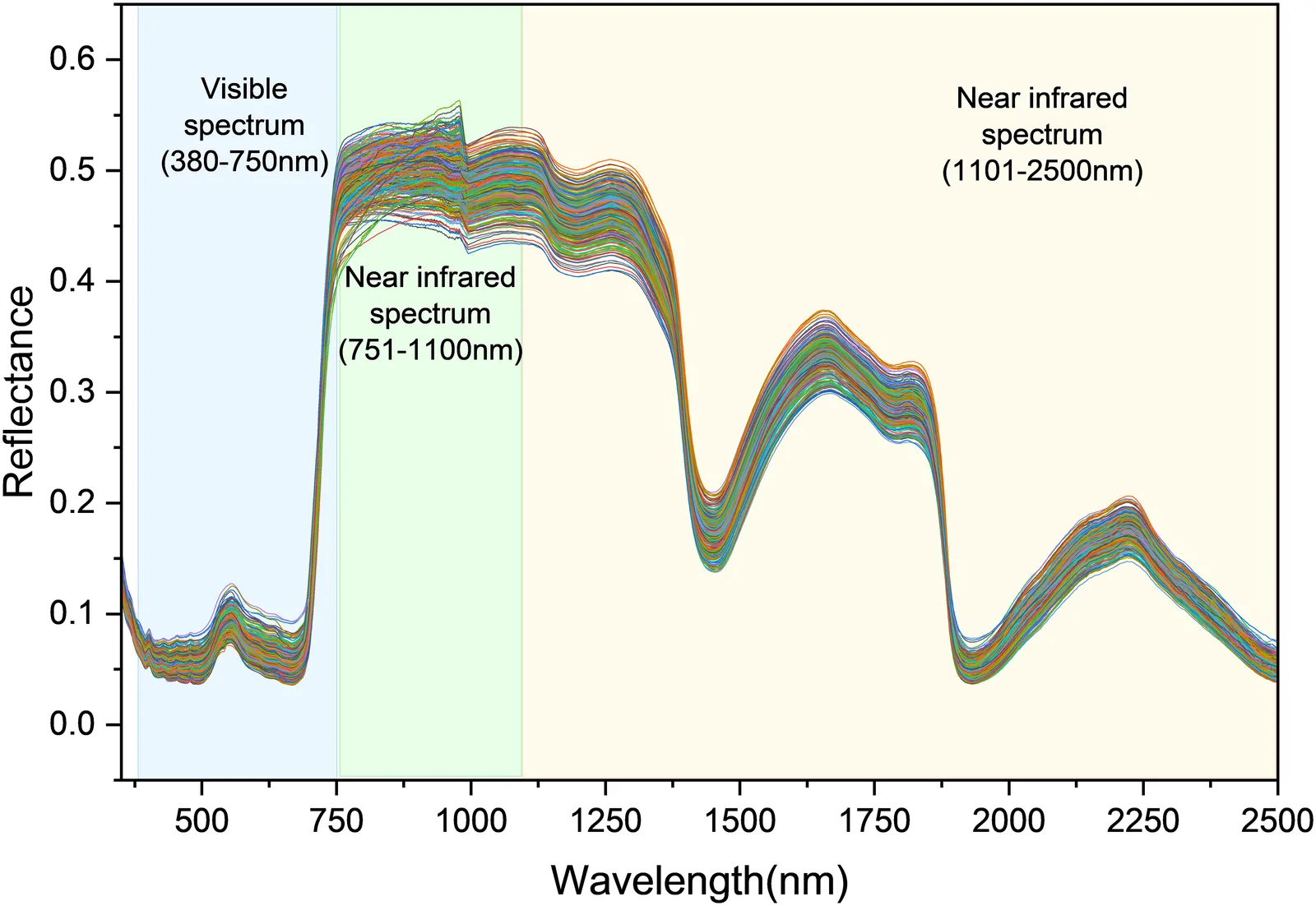
Spectral reflectance curves of all samples. The blue shaded region represents the visible spectrum (380–750 nm), the green shaded region represents the near-infrared spectrum (751–1000 nm), and the orange shaded region represents the short-wave infrared spectrum (1001–2500 nm).

Hyperspectral data from fig leaves were collected using an SR-3500 portable ground-based spectrometer (Spectral Evolution, USA), with a spectral detection range of 350–2500 nm (**Figures 4b**, **5**). The spectral resolution is 3 nm in the 350–1000 nm range, 8 nm at 1500 nm, and 6 nm at 2100 nm, providing a total of 2,151 spectral channels. Spectra were acquired *in vivo* on attached leaves using a leaf-clip accessory and a 25°field-of-view fiber-optic probe placed in direct contact with the adaxial (upper) leaf surface. Illumination was provided by the integrated halogen light source of the leaf clip, and measurement geometry was kept constant throughout the experiment. Considering the palmate structure of fig leaves, two sampling points were selected from each of the five lobes, while the major veins were intentionally avoided to minimize their influence on spectral measurements. Consequently, ten spectral measurements were collected from each leaf. The sampling points were distributed across different lobes to capture the overall spectral characteristics of the leaf rather than targeting only symptomatic or healthy regions. The average reflectance spectrum of these ten measurements was used as the representative spectrum of each leaf. Considering the high noise levels observed at the spectral edges, only wavelengths between 400 and 1000 nm were retained for subsequent analysis.

### 2.4 Data preprocessing

#### 2.4.1 Hyperspectral data preprocessing

Raw hyperspectral reflectance data were exported from the spectrometer using View Spec Pro (Analytical Spectral Devices, Colorado, US). Based on the characteristics of the spectral reflectance curves, noise generated by the instrument at the beginning and end of the spectrum was removed, and valid spectral data with low noise levels were retained for subsequent analysis. Spectral preprocessing methods, including Savitzky–Golay (SG), standard normal variate (SNV), the first derivative (FD), were applied. Specifically, SG smoothing was used to reduce random noise, the SNV algorithm was used to eliminate spectral scattering effects and baseline drift, and FD was used to enhance spectral details and improve the resolution of spectral peaks and valleys. The preprocessing methods were compared based on their spectral characteristics and five-fold cross-validation performance. The optimal preprocessing method was then selected for subsequent feature selection and model development.

#### 2.4.2 Selection of feature bands

The hyperspectral data for each sample consists of 2,151 spectral bands, containing substantial inter-band correlation and redundant information that could adversely affect the efficiency and accuracy of subsequent data processing and modeling. Therefore, dimensionality reduction and feature wavelength selection are essential for hyperspectral data analysis (Raghavendra et al., 2021). In this study, SPA and CARS were used for feature wavelength selection.

SPA projects the original spectral data into a low-dimensional space and optimizes feature selection by iteratively adjusting the projection direction. This algorithm effectively reduces redundant information while retaining informative variables, enabling the identification of key wavelengths that contribute to model performance and thereby improving predictive accuracy (Fangpeng et al., 2023). CARS simulates biological evolution to adaptively reweight and select spectral bands, gradually eliminating redundant and uninformative variables. This process reduces the influence of collinearity and improves the stability and robustness of the model (Li and Yang, 2023). Feature wavelength extraction was implemented in Python using PyCharm 2024.1.4 development environment.

### 2.5 Regression models

To establish a quantitative relationship between fig rust severity and hyperspectral feature variables, we selected three representative machine learning regression models—PLSR, SVR, and XGBoost—for modeling and analysis. Because lesion coverage is a continuous variable, stratified sampling was used to maintain a consistent sample distribution across different disease severity levels. The dataset was randomly divided at the leaf level into a training set (n = 268) and a held-out test set (n = 68) at a ratio of 8:2 using a fixed random seed (random_state = 42). Spectral preprocessing, feature selection (SPA and CARS), model optimization, and feature normalization were performed using only the training set, while the held-out test set was used exclusively for final model evaluation. To ensure a fair comparison among different modeling approaches, all regression algorithms were developed using the same training and test datasets. The data were randomly divided at the leaf level into a training set and a held-out test set. Because tree identities were unavailable, potential within-tree dependence across the two subsets cannot be excluded. The key parameter settings for spectral preprocessing, feature selection, regression modeling, and cross-validation are summarized in **Supplementary Table 1**.

#### 2.5.1 Partial least squares regression

PLSR is one of the most widely used linear modeling methods for quantitative spectral analysis, combining the advantages of multiple linear regression, canonical correlation analysis, and principal component analysis (Cheng and Sun, 2017). During model construction, the algorithm maximizes the covariance between the latent variables of the predictor matrix and the independent variables, thereby improving model accuracy and interpretability (Lin and Liu, 2022). In this study, the optimal number of latent variables was determined based on the minimum RMSECV obtained from the training dataset.

#### 2.5.2 Support vector regression (SVR)

SVR is a classic regression variant of the Support Vector Machine. Its objective is to identify an optimal regression function while allowing a controlled prediction error, thereby combining strong nonlinear modeling capability with good generalization performance (Dietrich et al., 1999). Unlike linear regression and decision tree regression, SVR does not attempt to fit every data point perfectly but instead relies on key support vectors, making it particularly suitable for regression problems involving small to medium-sized datasets and complex nonlinear relationships. In this study, the SVR model employed an RBF kernel. The penalty parameter (C), kernel coefficient (gamma), and insensitive loss parameter (epsilon) were set to 60, 0.01, and 0.01, respectively.

#### 2.5.3 Extreme gradient boosting regression (XGBoost)

XGBoost is a supervised gradient boosting algorithm proposed by Chen and Guestrin (2016) that uses the Newton method to optimize the loss function and incorporates a regularization term into the objective function. In generally, the algorithm improves predictive performance by iteratively generating new decision trees to fit the residuals of previous predictions, thereby reducing the discrepancy between predicted and observed values. Compared with traditional boosting algorithms, XGBoost provides better control of overfitting through regularization. In this study, the XGBoost model was configured with 300 estimators, a maximum tree depth of 3, a learning rate of 0.01, a subsampling ratio of 0.9, and a column sampling ratio of 0.6. The L1 and L2 regularization parameters were set to 1 and 2, respectively.

### 2.6 Accuracy testing

To comprehensively evaluate the predictive performance of the regression models, the coefficient of determination (R^2^), root mean square error (RMSE), and relative percentage deviation (RPD) were calculated. R^2^ measures a model’s ability to explain the variation in the dependent variable, with values closer to 1 indicating a better model fit. RMSE measures the deviation between the predicted and the observed values; smaller RMSE values indicate higher predictive accuracy. RPD evaluates the predictive reliability of the model, where SD represents the standard deviation of the observed values and SEP represents the standard error of prediction. An RPD value< 1.4 indicates poor predictive performance. Values between 1.4 and 2.0 indicate acceptable performance for rough estimation of the target variable, whereas values between 2.0 and 2.5 indicate good predictive performance suitable for quantitative prediction. An RPD value > 2.5 indicates excellent predictive performance suitable for practical applications (Hastie et al., 2009). The formulas are as follows (Equations 3–5):

(3) $$R^{2}=1-\frac{\sum_{i=1}^{m}{\left({\hat{y}}_{i}-{\bar{y}}_{i}\right)}^{2}}{\sum_{i=1}^{m}{\left(y_{i}-{\bar{y}}_{i}\right)}^{2}}$$

(4) $$RMSE=\sqrt{\frac{\sum_{i=1}^{m}{\left(y_{i}-{\hat{y}}_{i}\right)}^{2}}{m}}$$

(5) $$RPD=\frac{SD}{SEP}$$

## 3 Results

### 3.1 Analysis of changes in physiological parameters of figs

#### 3.1.1 Differences in physiological parameters across different severity levels

**Figure 6:**
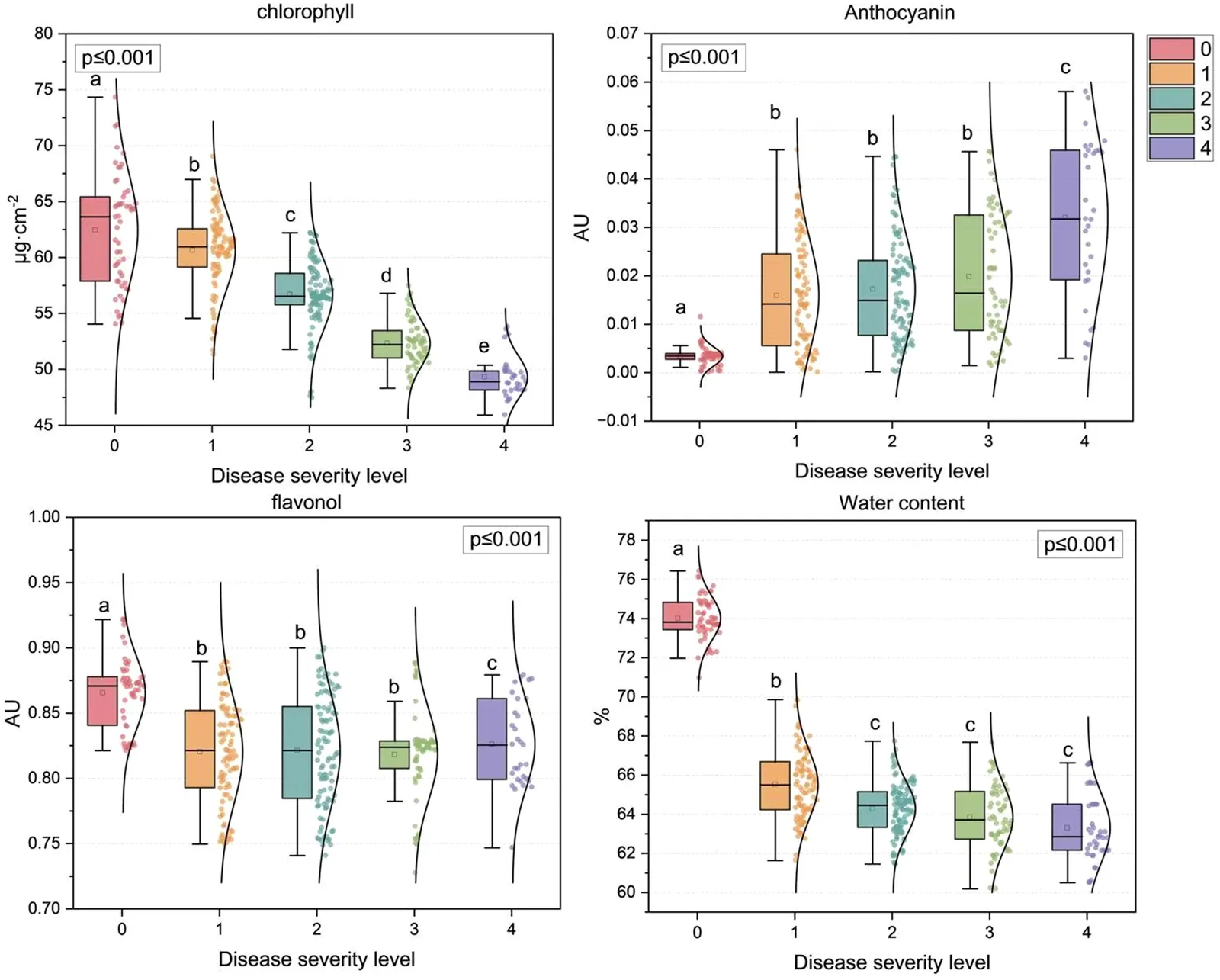
Box-and-whisker plots of chlorophyll, anthocyanin, flavonol, and leaf water content in fig leaves at different disease severity levels. Lowercase letters (a‑e) indicates significant‑difference grouping results from one‑way ANOVA. Different lowercase letters denote statistically significant differences (p ≤ 0.001) between disease‑severity levels.

As shown in **Figure 6**, to evaluate the effects of different fig rust severity levels on leaf physiological characteristics, box-and-whisker plots were generated for chlorophyll, anthocyanin, flavonol, and leaf water contents across five disease severity levels (0–4). One-way analysis of variance was performed to assess differences among the severity levels. The results showed significant differences in all physiological indicators among the different disease severity levels (p< 0.001). As disease severity increased, chlorophyll content showed a significant decreasing trend, whereas anthocyanin content increased progressively. Flavonol content fluctuated across the different disease severity levels, while leaf water content decreased continuously with increasing disease severity.

Overall, fig leaves at different disease severity levels exhibited significant differences in photosynthetic pigments, antioxidant-related compounds, and water status, providing a basis for the quantitative monitoring and grading of fig rust using physiological indicators.

#### 3.1.2 Analysis of the correlation between physiological parameters and disease

**Figure 7:**
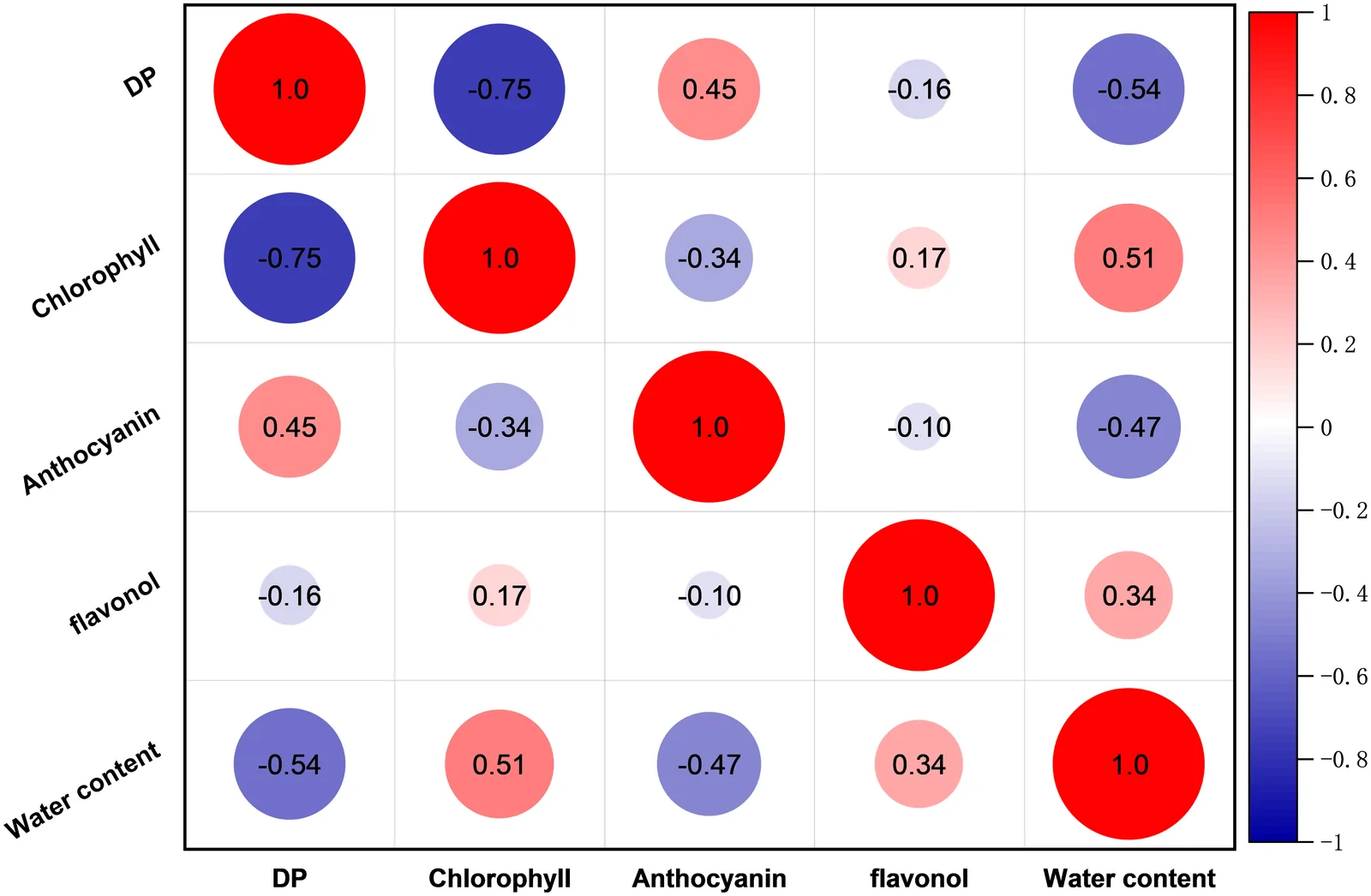
Heatmap showing the correlations between physiological parameters and fig rust severity.

Pearson correlation analysis was performed to quantify the relationships between fig rust severity (DP) and chlorophyll, anthocyanins, flavonols, and leaf water contents. Significance levels were set at p ≤ 0.05, p ≤ 0.01, and p ≤ 0.001. The results are shown in **Figure 7**. Lesion coverage showed a highly significant negative correlation with chlorophyll content (r = −0.75) and leaf water content (r = −0.54), a highly significant positive correlation with anthocyanin content (r = 0.45), and a weak negative correlation with flavonol content (r = −0.16). Among the physiological indicators, chlorophyll content was highly positively correlated with flavonol and leaf water content, whereas anthocyanin content was highly negatively correlated with leaf water content. No significant correlation was observed between anthocyanin and flavonol contents.

Overall, chlorophyll, anthocyanins, and leaf water contents showed stronger correlations with disease severity than flavonol content, suggesting that these physiological indicators have greater potential for quantitative disease monitoring.

### 3.2 Analysis of hyperspectral response characteristics of leaves at different disease severity levels

#### 3.2.1 Analysis of raw spectral characteristics

**Figure 8:**
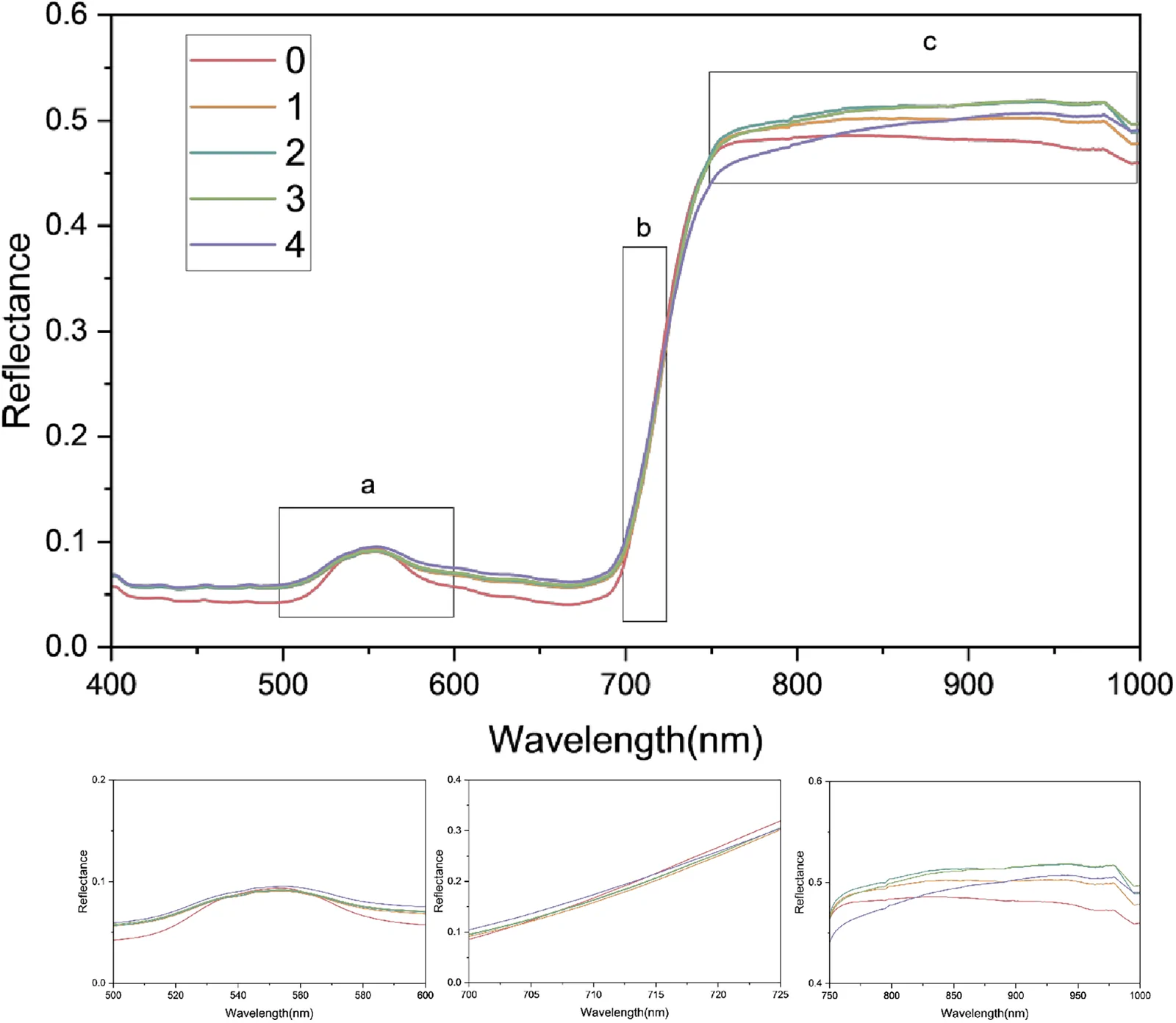
Mean spectral reflectance curves of fig leaves under rust severity levels 0–4. **(a)** Green spectral region; **(b)** red spectral region; **(c)** near-infrared spectral region.

**Figure 8** shows the mean spectral reflectance curves of fig leaves with different rust severity levels in the 400–1000 nm range. All spectra exhibited typical vegetation spectral characteristics. Reflectance was relatively low in the visible region (400–700 nm), with a distinct green peak near 550 nm. Compared with healthy leaves, rust-infected leaves generally exhibited higher reflectance in the visible region, and the differences became more pronounced with increasing disease severity. In the red-edge region (680–750 nm), reflectance increased sharply for all severity levels, while noticeable differences in the red-edge transition were observed among disease grades. In the near-infrared region (750–1000 nm), reflectance remained high and showed an increasing trend from Grade 0 to Grade 3, followed by a slight decrease at Grade 4.

Overall, rust infection altered the spectral response characteristics of fig leaves in both the visible and near-infrared regions, indicating that disease progression affected leaf physiological status and internal structure. These spectral differences provide a basis for subsequent sensitive wavelength selection and disease severity estimation.

#### 3.2.2 Analysis of spectral preprocessing results

**Figure 9:**
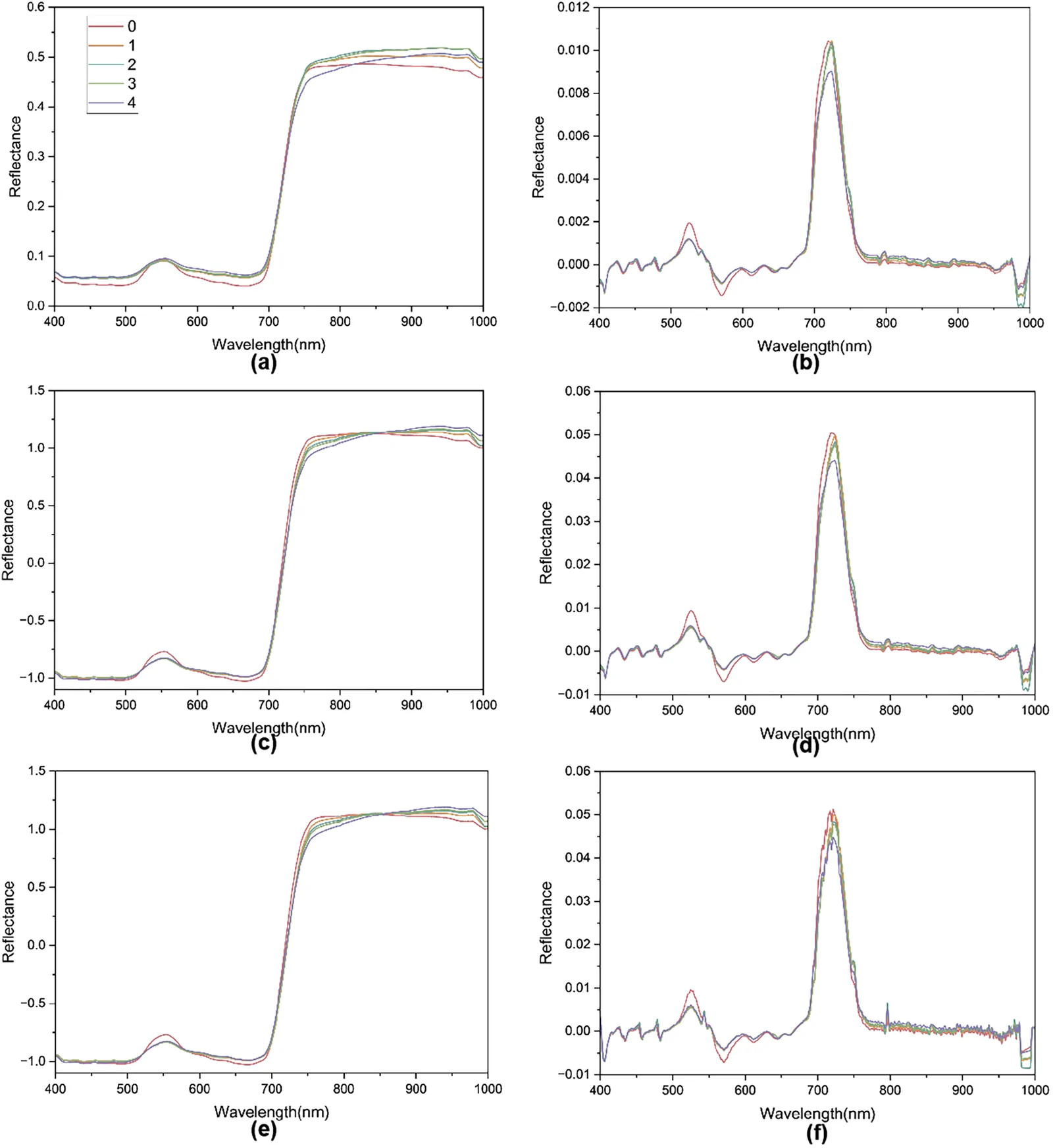
Spectral reflectance curves after different preprocessing methods. **(a)** SG; **(b)** SG+FD; **(c)** SG+SNV; **(d)** SG+SNV+FD; **(e)** SNV; **(f)** SNV+FD.

As shown in **Figure 9**, to reduce the effects of noise and scattering in the raw spectral data and improve the accuracy of model inversion, the spectral data were preprocessed using SG, SNV, and their combinations (SG+SNV, SG+FD, SNV+FD, and SG+SNV+FD). Overall, all preprocessing methods preserved the basic morphological characteristics of the vegetation spectra, although differences were observed in the spectral details. Among them, SG processing reduced effectively spectral noise, resulting in smoother curves. SNV processing reduced scattering effects to a certain extent but also amplified local fluctuations. After introducing the first-order derivative (FD), subtle spectral changes were further enhanced, making local disease-related spectral response characteristics more distinct.

**Figure 10:**
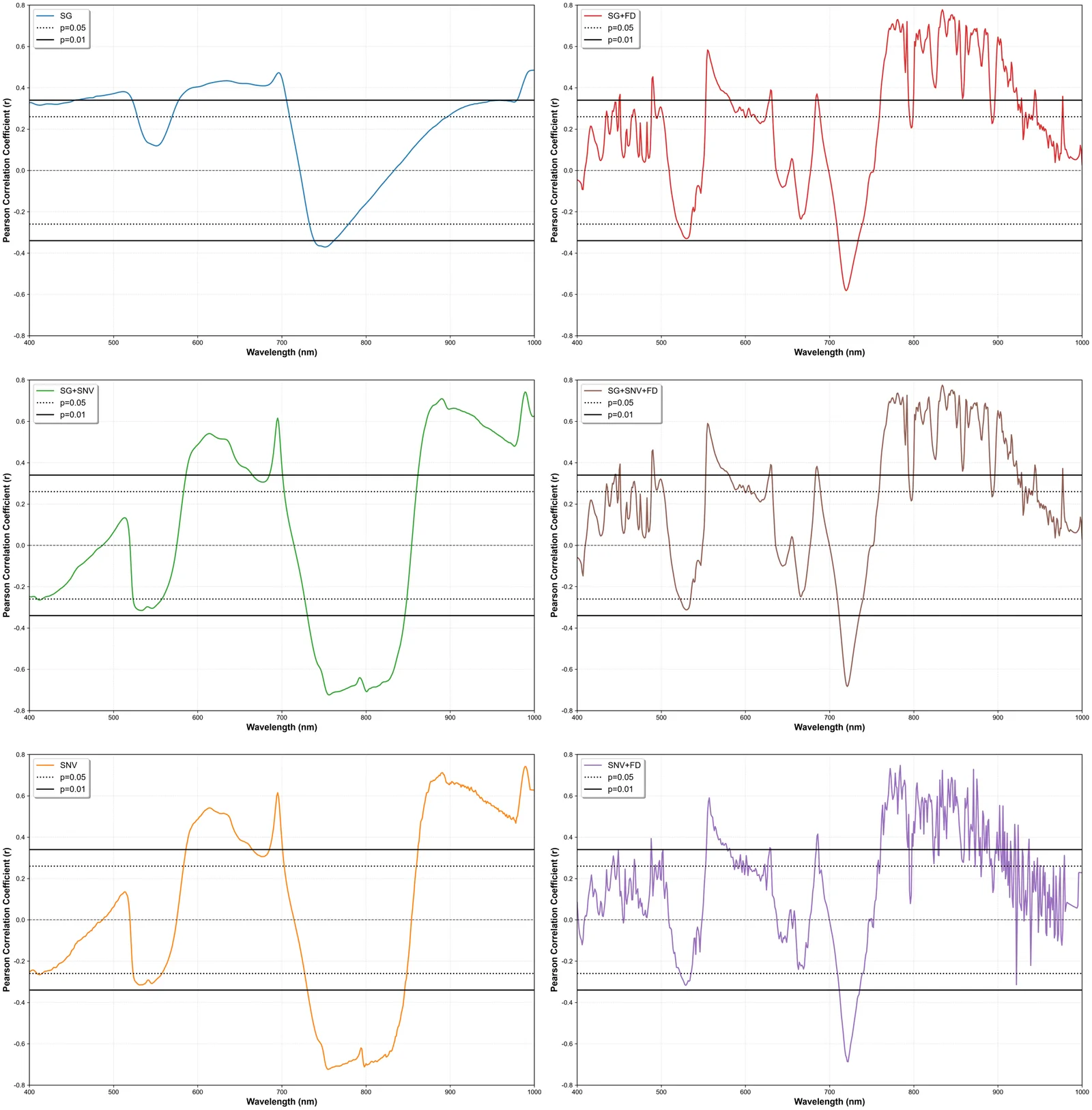
Correlation between different pretreatments and rust stress.

As shown in **Figure 10**, Pearson correlation analysis incorporating disease patch proportion (DP) showed that the correlation between spectral data and disease severity exhibited similar spatial patterns across the different preprocessing methods but differed in correlation strength and significance. In particular, in the visible red-edge transition region (approximately 680–750 nm) and parts of the near-infrared region, the SG+FD preprocessed spectra exhibited higher correlation stability and continuous response characteristics (p< 0.05 or p< 0.01), indicating that this combined method can more effectively enhance disease-sensitive information while suppressing redundant noise.

As shown in **Table 2**, different preprocessing methods exhibited noticeable differences in predictive performance. Among the six methods evaluated, SG+FD achieved the best results, with an average R^2^ of 0.821 ± 0.015, RMSE of 4.965 ± 0.153, and RPD of 2.371 ± 0.095 in five-fold cross-validation. In contrast, SG, SNV, SG+SNV, and SG+SNV+FD showed relatively lower predictive accuracy. These results indicate that SG+FD was the most effective preprocessing strategy for enhancing disease-related spectral information and was therefore selected for subsequent analyses.

**Table 2:** Performance comparison of different spectral preprocessing methods based on five-fold cross-validation.

Preprocessing method	R^2^ (Mean ± SD)	RMSE (Mean ± SD)	RPD (Mean ± SD)
SG	0.713 ± 0.011	6.296 ± 0.082	1.868 ± 0.037
SNV	0.719 ± 0.007	6.234 ± 0.048	1.887 ± 0.025
SG+SNV	0.721 ± 0.012	6.207 ± 0.082	1.895 ± 0.039
SG+FD	0.821 ± 0.015	4.965 ± 0.153	2.371 ± 0.095
SNV+FD	0.807 ± 0.007	5.169 ± 0.040	2.275 ± 0.043
SG+SNV+FD	0.780 ± 0.022	5.510 ± 0.251	2.138 ± 0.105

#### 3.2.3 Selection of spectral feature bands

After determining the optimal preprocessing method, SPA and CARS were employed to select feature bands from the preprocessed spectral data to reduce redundancy, improve computational efficiency, and enhance the representativeness of the selected features, thereby constructing an optimal subset of spectral variables.

(1) Results of SPA Feature Band Screening

**Figure 11:**
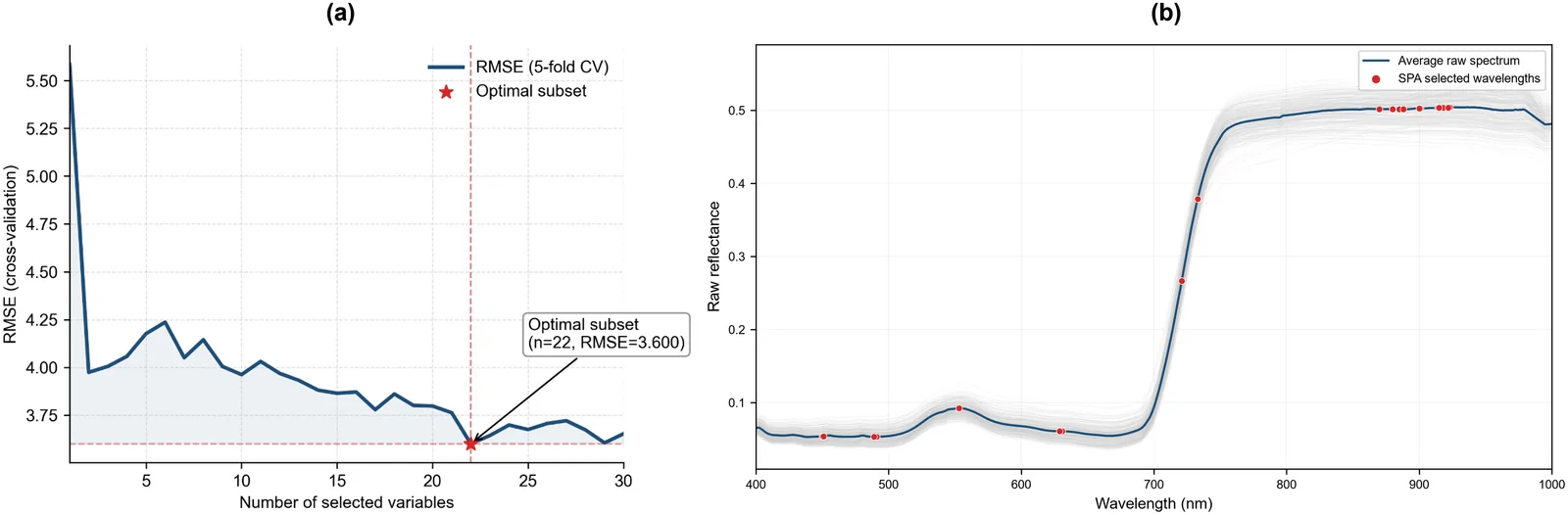
Feature wavelength selection using SPA. **(a)** Variation of 5‑fold cross‑validation RMSE versus the number of selected variables, red star represents the optimal subset. **(b)** Location of SPA‑selected characteristic wavelengths overlaid on the average raw reflectance spectrum.

The SPA algorithm reduces multicollinearity among variables by progressively introducing bands with the lowest correlation to the selected variables, thereby effectively eliminating redundant information. During model construction, the RMSECV from PLSR model was used as the evaluation metric to determine the optimal combination of variables. As shown in **Figure 11**, as the number of variables increased, the RMSECV generally decreased before reaching a stable level. When the optimal number of variables was reached, the RMSECV attained its minimum value. Based on the overall trend of the RMSECV curve, the optimal number of feature bands selected by SPA was determined to 22 (450, 451, 489, 490, 491, 553, 629, 631, 721, 733, 870, 880, 885, 886, 888, 900, 901, 914, 915, 918, 922, and 923 nm), primarily distributed within the 450–923 nm range. These results indicate that SPA can effectively reduce the dimensionality of the original spectral data while maintaining model prediction and accuracy.

(2) Results of CARS Feature Band Selection

**Figure 12:**
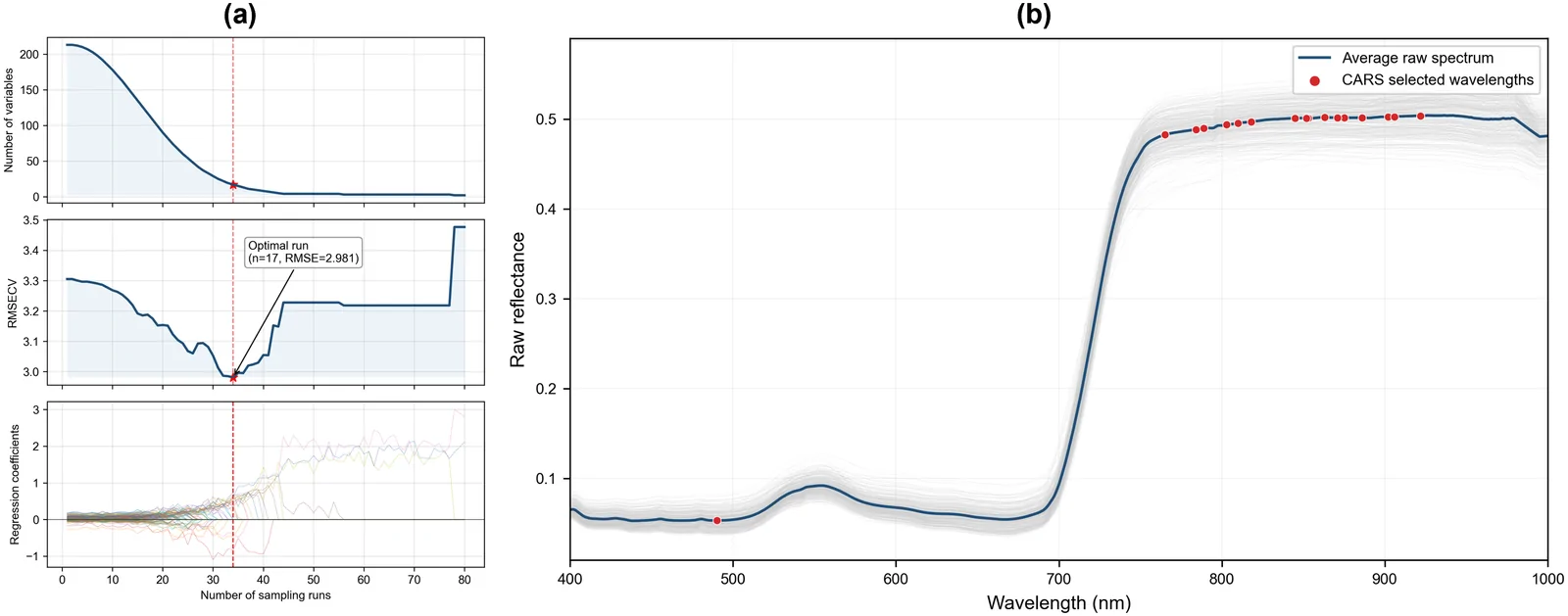
Feature wavelength selection using CARS. **(a)** The variation trend of the number of selected variables, RMSECV and regression coefficients with sampling runs, the red asterisk indicates the optimal sampling run (n=17, RMSECV=2.981). **(b)** Distribution of characteristic wavelengths selected by CARS overlaid on the average raw spectral reflectance curve.

The CARS algorithm iteratively selects variables using Monte Carlo sampling and an exponentially decreasing function and combines this process with the absolute values of the regression coefficients of the PLSR model to dynamically optimize the feature variables. This process gradually eliminates variables with minor contributions while retaining key spectral information that has substantial influences the target variable. As shown in **Figure 12**, during the iterative process, the number of variables decreased exponentially as the number of sampling iterations increased, whereas the RMSECV showed a fluctuating downward trend before stabilizing. At the 34th iteration, the RMSECV reached its minimum value, and the optimal subset consisted of 17 feature bands (490, 765, 784, 789, 803, 810, 818, 845, 852, 853, 863, 871, 875, 886, 902, 906, and 922 nm), primarily distributed within the 490–922 nm range.

### 3.3 Development and validation of a disease monitoring model

#### 3.3.1 Spectral-based disease monitoring model

**Figure 13:**
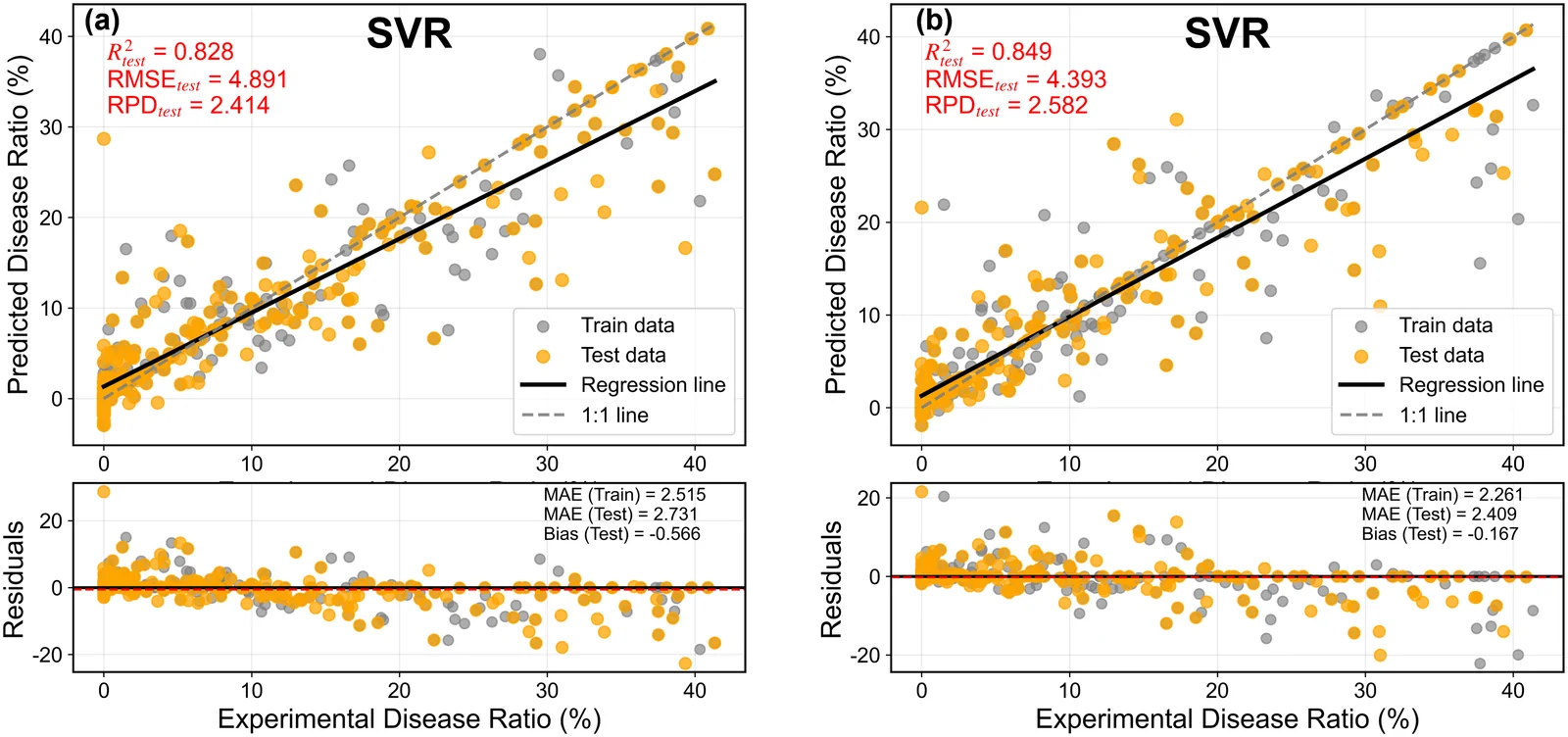
Performance of the optimal spectral-based disease severity estimation models using SPA and CARS. **(a)** Scatter plot and residual distribution of the SPA‑SVR model for disease ratio prediction; **(b)** Scatter plot and residual distribution of the CARS‑SVR model for disease ratio prediction.

Based on the characteristic wavelengths of SPA and CARS, disease severity models were constructed using SVR, PLSR, and XGBoost to evaluate the effects of different feature selection strategies on model performance. As shown in **Table 3** and **Figure 13**, prediction error generally increased with increasing lesion coverage, indicating a certain degree of heteroscedasticity in the models. In addition, for samples with severe disease, the SVR model tended to underestimate the disease severity, which may be related to the uneven distribution of samples across disease severity levels and the limited number of severely affected samples. Nevertheless, the CARS-SVR model achieved the best predictive performance (R^2^ = 0.849, RMSE = 4.393), providing a reference model for subsequent analyses integrating physiological parameters.

**Table 3:** Performance of spectral-based disease severity estimation models.

Feature selection	Model	Train set		Test set		
		R^2^	RMSE	R^2^	RMSE	RPD
SPA	SVR	0.859	4.384	0.828	4.891	2.414
	PLSR	0.724	6.138	0.720	6.264	1.888
	XGBoost	0.847	4.568	0.796	5.22	2.215
CARS	SVR	**0.861**	**4.351**	**0.849**	**4.393**	**2.582**
	PLSR	0.748	5.860	0.729	5.861	1.920
	XGBoost	0.840	4.666	0.803	5.230	2.253

Bold values represent the optimal prediction performance among all evaluated models.

#### 3.3.2 Physiological parameter-based disease monitoring model

**Figure 14:**
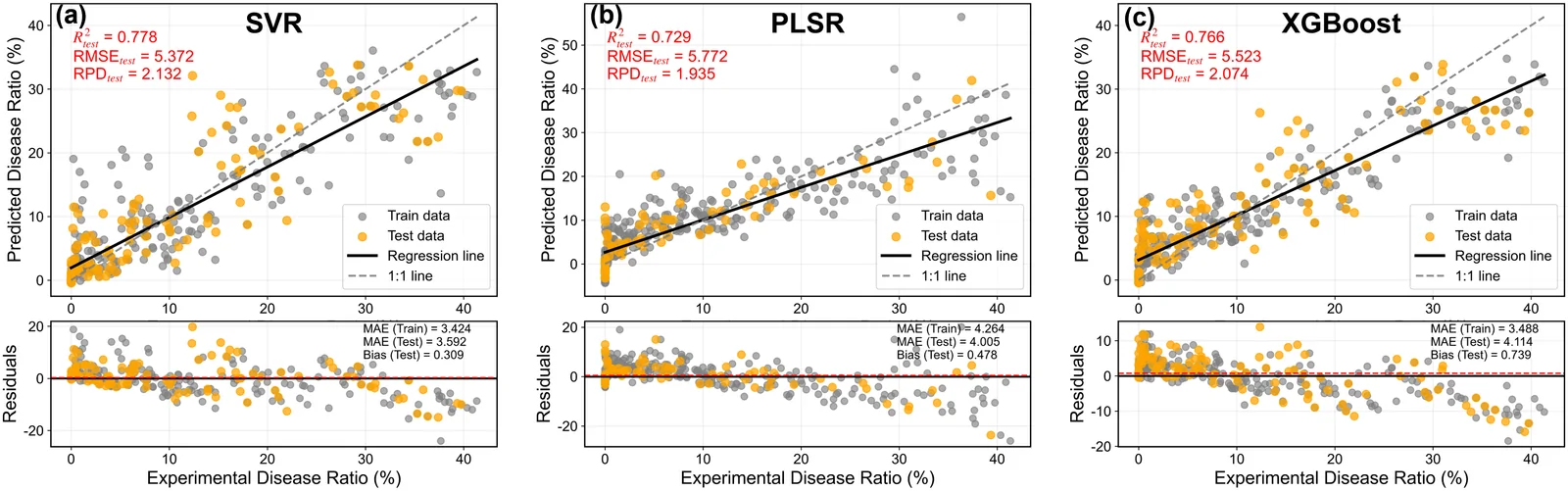
Performance of disease severity estimation models based on physiological parameters. **(a)** Scatter plot and residual distribution of the SVR prediction model; **(b)** Scatter plot and residual distribution of the PLSR prediction model; **(c)** Scatter plot and residual distribution of the XGBoost prediction model.

To evaluate the independent contribution of physiological information, chlorophyll, anthocyanin, and leaf water content were used as input variables for disease severity estimation. As shown in **Figure 14**, all models achieved moderate predictive performance. SVR provided the best results (R^2^ = 0.778, RMSE = 5.372, RPD = 2.132), followed by XGBoost and PLSR. Although physiological parameters reflected disease-induced changes in pigment composition and water status, their predictive performance remained lower than that of spectral-based models, indicating that physiological measurements alone cannot fully characterize the optical variations and spatial heterogeneity associated with rust development.

#### 3.3.3 Disease monitoring model combining spectral and physiological parameters

**Figure 15:**
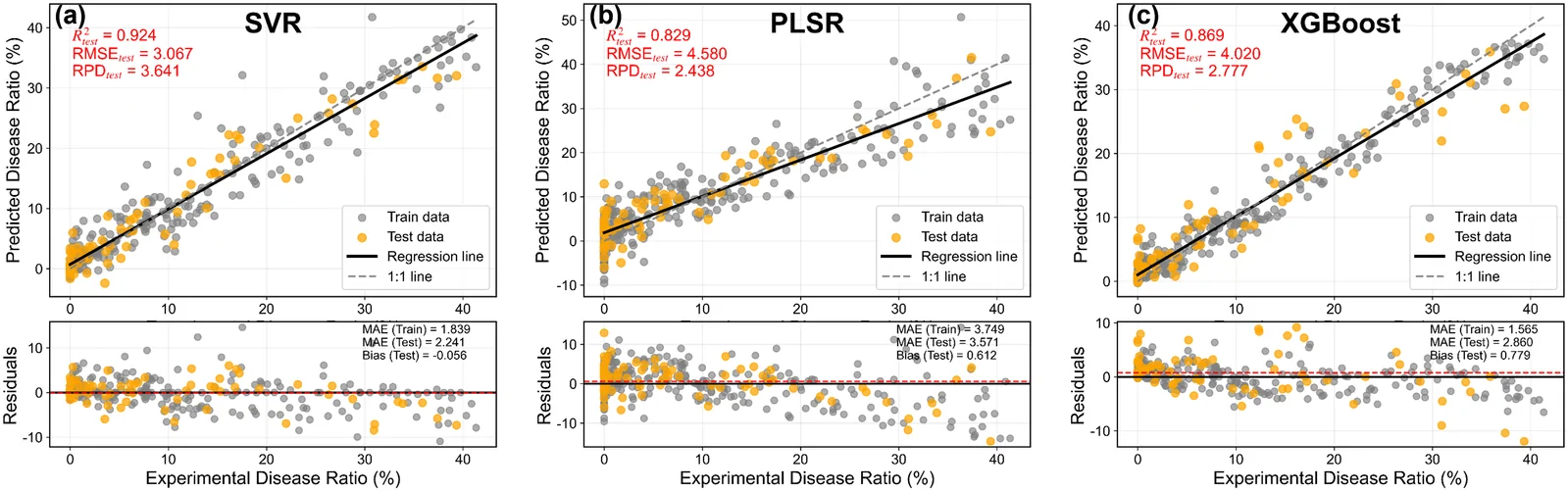
Disease severity estimation model integrating spectral and physiological parameters. **(a)** Scatter plot and residual distribution of the SVR prediction model; **(b)** Scatter plot and residual distribution of the PLSR prediction model; **(c)** Scatter plot and residual distribution of the XGBoost prediction model.

To investigate the complementary effects of physiological information, spectral features were integrated with chlorophyll, anthocyanin, and leaf water content to construct a fusion model. As shown in **Figure 15**, the fusion strategy showed better performance. The SVR model achieved the best performance (R^2^ = 0.924, RMSE = 3.067, RPD = 3.641), followed by XGBoost (R^2^ = 0.869) and PLSR (R^2^ = 0.829). Compared with the optimal spectral-only model (CARS-SVR), the fusion SVR model increased R^2^ by 8.8% and reduced RMSE by 30.2%, demonstrating that physiological parameters provided complementary information for disease severity estimation.

#### 3.3.4 Ablation analysis of physiological information contribution

As shown in **Table 4**, to further investigate the contribution of individual physiological variables and verify the complementary effects between spectral and physiological information, ablation experiments were conducted using the optimal CARS-SVR framework. Compared with the spectral-only model (R^2^ = 0.849), incorporation of chlorophyll showed better performance, increasing the test R^2^ to 0.911 and reducing RMSE from 4.393 to 3.311. This demonstrated that chlorophyll provided critical physiological information associated with rust-induced degradation of photosynthetic pigments. In contrast, the addition of anthocyanin or leaf water content individually resulted in lower predictive performance than that of the spectral-only model. The corresponding test R^2^ values were 0.802 and 0.823, respectively, suggesting that these variables did not provide additional predictive benefit when used individually within the current modeling framework. Among all tested combinations, the model integrating spectral features with chlorophyll, anthocyanin, and leaf water content achieved the highest predictive performance (R^2^ = 0.924, RMSE = 3.067, RPD = 3.641). Overall, the ablation results indicate that chlorophyll was the most informative physiological variable for fig rust severity estimation, whereas the individual contributions of anthocyanin and leaf water content were comparatively limited.

**Table 4:** Performance of spectral-based disease severity estimation models.

Model	Input variables	R^2^test	RMSEtest	RPD
SVR	CARS bands	0.849	4.393	2.582
	CARS bands + Chlorophyll	0.911	3.311	3.349
	CARS bands + Anthocyanin	0.802	4.934	2.248
	CARS bands + Water content	0.823	4.663	2.378
	CARS bands + Chlorophyll + Anthocyanin + Water content	0.924	3.067	3.641

## 4 Discussion

### 4.1 Changes in physiological parameters under rust stress and their response mechanisms

Pathogen infection typically begins by disrupting chloroplast structure and inhibiting the synthesis of photosynthetic pigments, leading to a decrease in chlorophyll content. At the same time, plants enhance their antioxidant capacity by inducing secondary metabolic pathways, causing dynamic changes in compounds such as anthocyanins (Chalker‐Scott, 1999). Li N. et al. (2022) on pine wilt disease showed that chlorophyll a, chlorophyll b and water contents in infected pine trees all decreased as the infection progressed. Similarly, Hornero et al. (2021) found that model-inferred plant traits in oaks affected by *Phytophthora cinnamomi* were significantly lower than those in healthy trees. The results of the present study showed that, as disease severity increases, chlorophyll and leaf water contents decreased significantly, whereas anthocyanin content increased significantly. Flavonol content fluctuated across different severity levels and showed only a weak correlation with lesion coverage. These findings characterize the progressive physiological changes in fig leaves under rust stress, including declines in photosynthetic function and leaf water status, together with stress induced secondary metabolism, and provide a physiological basis for the quantitative estimation of fig rust severity using hyperspectral remote sensing.

### 4.2 Analysis of spectral response mechanisms and methods for selecting characteristic wavelength bands

Hyperspectral reflectance is highly sensitive to physiological and structural changes induced by plant diseases. In this study, leaf reflectance increased in the visible region with increasing rust severity, whereas reflectance in the near-infrared region exhibited a trend of first increasing and then slightly decreasing. These changes may be related to chlorophyll degradation, variations in leaf water status, and structural alterations of mesophyll tissues during disease development (Mahlein et al., 2012). However, hyperspectral data contain substantial redundant and highly correlated spectral information; therefore, feature wavelength selection is crucial (Siedliska et al., 2018). This study applied both SPA and CARS to extract characteristic wavelengths for fig rust. The characteristic bands selected by SPA were mainly distributed within 450–631 nm and 721–923 nm, whereas those selected by CARS were concentrated at 490 nm and 765–922 nm. These results are highly consistent with the near-infrared sensitive bands (750–900 nm) reported by Deng et al. (2019) in their study of citrus huanglongbing, suggesting that the near-infrared region exhibits a common response to disease-induced damage to leaf structure. Modeling results further showed that CARS outperformed SPA, with the CARS-SVR model achieving the best predictive performance (R^2^ = 0.85, RMSE = 4.39). These findings indicate that the choice of feature selection algorithm has a substantial influence on the performance of spectral estimation models for plant diseases. Therefore, a universal feature selection strategy cannot be directly applied; instead, different algorithms should be evaluated according to the spectral characteristics of the target crop to identify the most suitable feature selection strategy.

### 4.3 Applicability of modeling methods and advantages of multi-source information fusion

Significant differences were observed among the machine learning algorithms in estimating disease severity. SVR achieved the best performance (R^2^ = 0.924, RMSE = 3.067, RPD = 3.641), followed by XGBoost and PLSR. This may be attributed to the strong capability of SVR to model nonlinear relationships in high-dimensional datasets under limited sample conditions (Dietrich et al., 1999). In contrast, PLSR is based on linear assumptions and therefore struggles to capture the complex nonlinear relationships between spectral data and disease severity (Yuan et al., 2014). Although XGBoost has strong nonlinear fitting capability, its performance may fluctuate when the sample size is limited (Chen et al., 2020). Furthermore, spectral information and physiological parameters provide complementary information, spectral data reflect external optical responses, whereas physiological parameters directly characterize internal biochemical states (Ustin and Gamon, 2010). Among the evaluated physiological variables, chlorophyll showed better performance, increasing the predictive accuracy from R^2^ = 0.849 to R^2^ = 0.911 when combined with spectral features. This finding is consistent with the observed decline in chlorophyll content during disease progression and highlights the close association between photosynthetic pigment degradation and rust severity. In contrast, anthocyanin and leaf water content did not improve model performance when incorporated individually into the spectral model. This suggests that the information contained in these variables may already be partially represented by the selected spectral features or may exhibit more complex relationships with disease development. Nevertheless, the model integrating spectral features with all physiological parameters achieved the highest overall performance (R^2^ = 0.924). Therefore, the combination of spectral and physiological information appears to provide a more comprehensive characterization of disease-induced changes in fig leaves than either information source alone.

### 4.4 Limitations of the study and future directions

Although this study achieved satisfactory performance in quantitative estimation of fig rust severity, several limitations should be acknowledged. First, prediction performance was not uniform across the full range of disease severity, with prediction errors generally increasing with lesion coverage and highly infected leaves tending to be underestimated. In addition, the dataset was collected from a single greenhouse during one disease development period, and the current model was not externally validated using datasets from different locations, cultivars, seasons, or environmental conditions. Therefore, the generalization capability and predictive reliability of the proposed model require further evaluation with larger and more diverse datasets. Second, this study focused on leaf-scale disease severity estimation based on hyperspectral and physiological information. Although the results demonstrate the potential of the proposed framework, direct application to orchard-scale disease monitoring has not yet been validated. Future studies should integrate UAV platforms and multi-source remote sensing data to investigate the feasibility of large-scale disease assessment under field conditions. Third, some physiological parameters, such as leaf water content, were obtained through destructive laboratory measurements, which limits their direct application in rapid field monitoring. Future research should explore non-destructive estimation approaches for physiological traits to further improve the practicality of the proposed framework. Finally, although approximately three to four functional leaves were collected from each tree, including leaves from different canopy positions, tree identity information was not recorded during the original sampling process. Therefore, the current dataset was randomly partitioned at the leaf level, and potential correlations among samples from the same tree cannot be completely excluded. Future studies should record tree-level information during sampling and adopt grouped validation strategies to provide a more rigorous assessment of model robustness.

## 5 Conclusion

Based on hyperspectral reflectance and physiological parameters of fig leaves, this study systematically investigated the physiological responses, spectral characteristics, and quantitative estimation of fig rust severity. The main conclusions are as follows:

1. Fig rust infection induced significant physiological changes in leaves. Chlorophyll and leaf water content decreased significantly with increasing disease severity, whereas anthocyanin content increased significantly. In contrast, flavonol content fluctuated among disease severity levels and showed only a weak correlation with lesion coverage. These results indicate that chlorophyll, anthocyanin, and leaf water content are more sensitive indicators of rust progression.
2. Among the evaluated preprocessing methods, SG+FD achieved the best performance. CARS outperformed SPA in feature wavelength selection and extracted 17 characteristic bands mainly distributed in the 490 nm pigment absorption region and the 765–922 nm near-infrared region. The optimal spectral model (CARS-SVR) achieved an R^2^ of 0.849 and RMSE of 4.393.
3. Physiological parameters alone provided moderate predictive capability (R^2^ = 0.778), whereas integrating physiological and spectral information substantially improved disease severity estimation. The fusion SVR model achieved the best performance (R^2^ = 0.924, RMSE = 3.067, RPD = 3.641). Ablation analysis further revealed that chlorophyll contributed most to model improvement, while the combination of multiple physiological parameters yielded the highest prediction accuracy.

Overall, the integration of hyperspectral and physiological information provides a promising approach for leaf-scale quantitative estimation of fig rust severity. However, predictive reliability decreased at high lesion coverage, with highly infected leaves tending to be underestimated, indicating that model performance was not uniform across disease severity levels. Thus, the proposed framework represents a leaf-level proof-of-concept that requires further validation using larger and more diverse datasets.

## Data Availability

The raw data supporting the conclusions of this article will be made available by the authors, without undue reservation.
