# Supplementary material for: Integrating hyperspectral features and physiological parameters for quantitative estimation of fig rust severity
Source: Front Plant Sci. 2026 Sep 11;17:1930419. doi: 10.3389/fpls.2026.1930419 (PMC13613503; doi:10.3389/fpls.2026.1930419)
Supplement: Supplementary file 1 [file Table1.docx]

Supplementary Table S1. Key parameter settings for spectral preprocessing, feature selection, regression models, and validation.

| Method | Key parameter settings |
| --- | --- |
| SG | Window length = 11; polynomial order = 2 |
| FD | First derivative; Savitzky–Golay method |
| SPA | Candidate variables = 2–30; selection by minimum RMSECV |
| CARS | Max runs = 80; 5-fold CV; decay rate = 0.08; |
| PLSR | Latent variables = 1–20; selection by minimum RMSECV |
| SVR | RBF kernel; C = 60; γ = 0.01; ε = 0.01 |
| XGBoost | 300 trees; max depth = 3; learning rate = 0.01; subsample = 0.90; colsample = 0.60; L1 = 1; L2 = 2 |
| Validation | 80:20 train//held-out test set split; 5-fold CV; random state = 42 |
| Software | Python 3.10, PyCharm 2024.1.4, ImageJ 1.53q |
